# Cumulative radiation dose from medical imaging in paediatric congenital heart disease patients with epicardial cardiac implantable electronic devices

**DOI:** 10.1093/ehjimp/qyae060

**Published:** 2024-06-13

**Authors:** Oluyemi B Aboyewa, Christina Laternser, Andrada Popescu, Nicole Murphy, Dhaivat Shah, Michael C Monge, Cynthia K Rigsby, Laleh Golestanirad, Gregory Webster, Daniel Kim

**Affiliations:** Department of Biomedical Engineering, Northwestern University, 2145 Sheridan Road, E310, Evanston, IL 60208, USA; Department of Radiology, Feinberg School of Medicine, Northwestern University, 737 N. Michigan Avenue Suite 1600, Chicago, IL 60611, USA; Division of Cardiology, Department of Pediatrics, Ann & Robert H. Lurie Children’s Hospital, 225 E Chicago Avenue, Chicago, IL 60611, USA; Department of Medical Imaging, Ann & Robert H. Lurie Children’s Hospital, 225 E Chicago Avenue, Chicago, IL 60611, USA; Department of Medical Imaging, Ann & Robert H. Lurie Children’s Hospital, 225 E Chicago Avenue, Chicago, IL 60611, USA; Division of Cardiology, Department of Pediatrics, Ann & Robert H. Lurie Children’s Hospital, 225 E Chicago Avenue, Chicago, IL 60611, USA; Division of Cardiovascular Surgery, Department of Surgery, Ann & Robert H. Lurie Children’s Hospital, 225 E Chicago Avenue, Chicago, IL 60611, USA; Department of Medical Imaging, Ann & Robert H. Lurie Children’s Hospital, 225 E Chicago Avenue, Chicago, IL 60611, USA; Department of Biomedical Engineering, Northwestern University, 2145 Sheridan Road, E310, Evanston, IL 60208, USA; Department of Radiology, Feinberg School of Medicine, Northwestern University, 737 N. Michigan Avenue Suite 1600, Chicago, IL 60611, USA; Division of Cardiology, Department of Pediatrics, Ann & Robert H. Lurie Children’s Hospital, 225 E Chicago Avenue, Chicago, IL 60611, USA; Department of Biomedical Engineering, Northwestern University, 2145 Sheridan Road, E310, Evanston, IL 60208, USA; Department of Radiology, Feinberg School of Medicine, Northwestern University, 737 N. Michigan Avenue Suite 1600, Chicago, IL 60611, USA

**Keywords:** radiation dose, epicardial leads, cardiac implantable electronic devices, congenital heart disease, paediatrics

## Abstract

**Aims:**

To determine whether paediatric congenital heart disease (CHD) patients with epicardial cardiac implantable electronic devices (CIEDs) receive high cumulative effective doses (CEDs) of ionizing radiation from medical imaging tests.

**Methods and results:**

We compared 28 paediatric CHD patients with epicardial CIEDs (cases) against 40 patients with no CIED matched by age at operation, sex, surgical era, and CHD diagnosis (controls). We performed a retrospective review of radiation exposure from medical imaging exams between 2006 and 2022. Radiation dose from computed tomography (CT) and X-ray radiography was calculated using the National Cancer Institute Radiation Dosimetry Tool. We performed univariate analysis to compare the CED between the two groups. In the case subgroup, we convened experts’ review to adjudicate the prevalence of CT exams that should have been performed with magnetic resonance imaging (MRI) in the absence of a CIED. Children (median age 2.5 years at implant) with CIEDs received significantly higher median CED compared with matched controls (6.90 vs. 1.72 mSv, *P* = 0.0018). In cases, expert adjudication showed that 80% of the CT exams would have been performed with MRI in the absence of a CIED. This resulted, on average, a five-fold increase in the effective dose (ED) from post-lead implant CTs.

**Conclusion:**

Paediatric CHD patients with CIED received four times higher CED than matched controls. Improved access to medical imaging tests without ionizing radiation, such as MRI, could potentially reduce the ED in CIED patients by up to five times.

## Introduction

Congenital heart disease (CHD) is the most common disease in children that requires a pacemaker or implantable defibrillator. These devices are collectively referred to as cardiac implantable electronic devices (CIEDs). Most CIEDs consist of two main components: the pulse generator (battery and computing system) and the leads (wires that connect to the heart). The preferred approach in adolescents and adults, if anatomy allows, is to pass one or more leads through the veins and affix them to the inside of the heart (‘endocardial leads’). The standard of care for young children and people with complex venous anatomy is to affix leads to the heart by opening the chest and sewing them directly to the myocardium (‘epicardial leads’).^1,2^ The global CIED market is projected to reach USD 28.8 billion by 2030^3^ with an increasing trend in their utilization in children.^4^

It has been estimated that 50–75% of patients with a CIED would require magnetic resonance imaging (MRI) over the lifetime of the device.^5^ Indeed, MRI is a critical modality for CHD patients who require frequent monitoring following corrective surgeries; it offers excellent tissue contrast without ionizing radiation. While there are CIEDs with endocardial leads that are magnetic resonance (MR)–conditional, there are no CIEDs with epicardial leads that are MR-conditional. A device is labelled MR-conditional if it is used in a specified MR environment and conditions in which it does not pose a known hazard; otherwise, it is labelled non–MR-conditional.^6^ There are theoretical risks that non–MR-conditional systems could cause tissue heating, damage, or arrhythmias in the MR environment.^7^ To date, MRI data are extremely limited for paediatric patients, with current publications including fewer than 500 children with a CIED in total.^6,8–11^

The 2017 Heart Rhythm Society expert consensus statement made no recommendation for MRI in paediatric population with epicardial leads.^6^ The 2021 Pediatric and Congenital Electrophysiology Society (PACES) guideline made a Class IIb recommendation for MRI for paediatric CIED patients with epicardial or abandoned leads on an individualized consideration based on the risk/benefit ratio. However, few data exist to quantify those risks and benefits.^12^ Not all children’s hospitals are equipped to make informed decisions on the risk/benefit ratio. In the absence of supporting data, many centres default to imaging modalities with ionizing radiation in non–MR-conditional CIED systems, especially computed tomography (CT). Ionizing radiation from medical imaging modalities such as CT, X-ray (XR), fluoroscopy, and nuclear imaging increases the risk of cancer.^6,8,9,13^ Children have higher cancer risks from ionizing radiation than adults because developing bodies are more sensitive to ionizing radiation.^14^

To our knowledge, there are no data comparing the cumulative radiation exposure from medical imaging tests between paediatric CHD patients with and without a CIED. The purposes of this retrospective study were to determine if children with CHD and CIEDs (cases) receive increased ionizing radiation compared with CHD patients with no CIED (controls), and whether avoidable CTs in the absence of a CIED result in a significant decrease in cumulative radiation exposure. An increased ionizing radiation burden in childhood would highlight an unmet need to increase MRI access for children with CIEDs and directly inform the risk–benefit calculation stipulated in the 2021 PACES guidelines.

## Methods

### Patient selection

**Figure 1:**
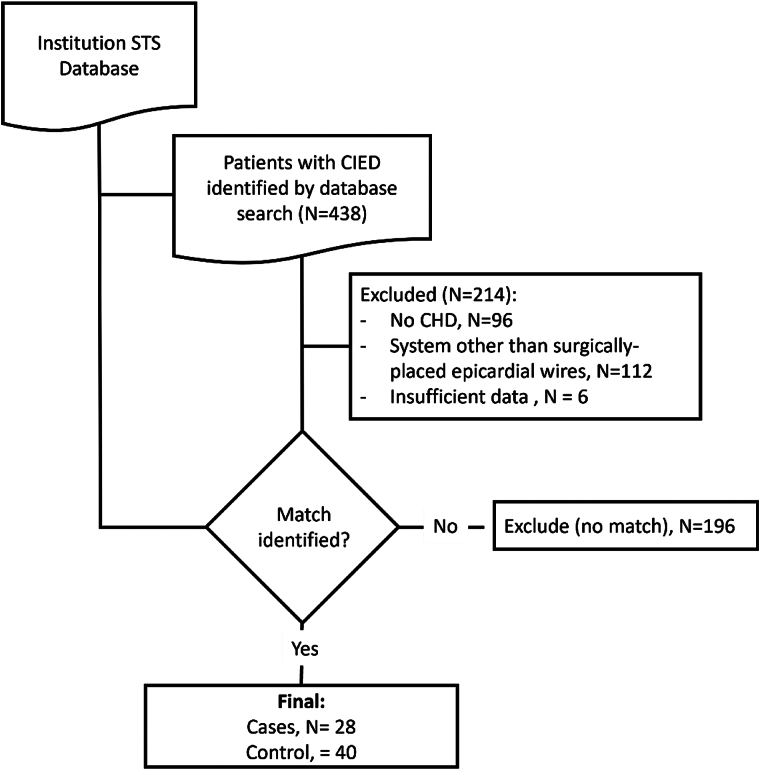
Patient selection for inclusion in the study. We identified patients matched by age at operation, sex, surgical era, and CHD diagnosis from our institution’s implementation of the STS national database. Twenty-one of the 28 cases had identified matches while 7 had reasonable matches in the control group.

We performed a retrospective review of children with CHD (October 2006–June 2022) at a tertiary paediatric heart centre. For inclusion (*Figure 1*), all patients were required to have a non–MR-conditional epicardial CIED (*n* = 438). We excluded patients with endocardial systems and no epicardial leads, regardless of MR-conditional status. We used our local implementation of the Society of Thoracic Surgeons (STS) National Database to identify patients matched by age at operation, sex, surgical era, and CHD diagnosis who did not receive a CIED. We first matched patients by diagnosis, using a comparison of the ‘Fundamental Diagnosis’ field in the STS Database, followed by a detailed review of anatomic data in the electronic medical record to ensure accurate matching of the exact diagnosis, not just general classification. That is, the diagnosis was matched on a strict anatomic basis (no anatomic categories were used to simplify matching). We further matched on age at operation and sex. We then required that each patient was matched with a patient in the same surgical era, which was defined as a surgery occurring within 2 years in either direction of the operative date for the patient selected as a case.

Follow-up duration was defined from the first date of clinical care at our centre until the last date of clinical care, censored at age 17 years for both cohorts. Patients’ Digital Imaging and Communication in Medicine (DICOM) files were de-identified by the hospital's information technology team and used for analysis. The institutional review board at our institution exempted this study from human subjects protection.

### Effective dose calculation

Effective dose (ED) is a common biological risk metric from ionizing radiation, measured in Sieverts (Sv). ED is suitable for comparing relative radiation risk across medical imaging modalities, patient populations, age, sex, and body habitus.^15^ ED calculates a weighted sum of the mean absorbed dose of radiation in all organs or tissues while considering the radiation type’s relative biological effectiveness or harm level. Because ED represents the stochastic health risk to the whole body regardless of the organ or tissue irradiated, it enables summation of radiation dose across different exams or body parts.

Organ dose can be estimated by embedding dosimeters, such as thermoluminescent dosimeters (TLDs), within a physical phantom or by simulation in virtual or computational phantoms.^16^ Computational phantoms (such as VirtualDose, CT-Expo, and ImPACT) incorporate radiation transport codes with an anatomic model of the patient to account for dose distribution and are increasingly used for ED calculations.^17^ CT doses calculated using the publicly available National Cancer Institute (NCI) dosimetry software have been shown to align with TLD values within 8–20%, particularly in infants and children,^18–20^ and produce similar results to other virtual phantoms.^21^ Therefore, for this retrospective study, all ED doses were calculated using the NCI dosimetry software.

For each patient, the ED from each ionizing radiation exposure was summed to calculate the cumulative effective dose (CED). There were no radiation therapies or other non-cardiac therapeutic exposures to consider among our cohort. For the primary statistical analysis of CED, we used data from all patients. For the secondary analysis, we used data from a subgroup of patients to evaluate CED exposure from CT exams among patients who received at least one CT exam. Radiation dose from interventional fluoroscopy and catheterization was not included in the CED calculations.

#### CT dose

The CED from each CT exam was calculated using the NCI dosimetry system for CT (NCICT, version 3.0 ).^22^ Relevant scan parameters such as tube potential (kVp), tube current (mA), rotation time (s), CT dose index (CTDI_vol_), scan length, spiral pitch factor, and collimation (mm) along with scanner vendor and model information were obtained from the CT scanner protocol and DICOM file header for each patient (see Supplementary data online, *Figure S1A*).

The NCICT software calculates organ doses in the International Commission on Radiological Protection (ICRP) paediatric and adult reference computational phantoms using dose coefficients derived from Monte Carlo simulation of XR interactions in a reference CT scanner.^22–24^ Pre-defined NCICT software age groups were used to select male and female paediatric phantoms: <10 months, 10 months–2 years, 3–7 years, 8–12 years, and 13–17 years.^25^ To simplify the CED calculations in the software, we assumed no tube current modulation,^26^ with the same phantom sizes selected for both patient groups.

#### XR dose

The NCI dosimetry system for Radiography and Fluoroscopy (NCIRF, version 2.0 )^27^ was used to calculate CED for each XR of the chest, full-body infant radiographs, and abdomen (See Supplementary data online, *Figure S1B*). It combines the ICRP paediatric and adult reference computational phantoms with a streamlined GEANT4 Monte Carlo radiation transport code.^28^ Organ dose calculations were made in the pre-defined five age groups for male and female paediatric phantoms, the same as those used in the CT dose calculation. Software parameters for ED calculation include XR beam energy kVp, half-value layer (HVL; mm Al), source-to-isocentre distance, isocentre, field of view (FOV; cm^2^), beam direction, and dose area product (DAP; Gy cm^2^).

To simplify the XR CED calculation across 68 eligible patients (i.e. total XRs = 3685), representative parameter lookup tables including kVp, mAs, and DAP were created for the five phantom age groups by randomly selecting 10 patients in the database who had a single view [anteroposterior (AP)] and two views (AP and lateral) of XRs of the chest, full-body, and abdominal exams. The average DAP, and the combination of kVp and HVL that most closely matched those in the NCIRF dropdown menu options, was selected for the dose calculation (see Supplementary data online, *Tables S1* and *S2*). Also, all dose calculations were made with the phantom hands raised, excluding the arm region from the FOV of interest and at a source-to-isocentre distance of 100 cm. This models our clinical patient set-up. The patient’s reported CED was calculated by multiplying the number of XR exams with the mean ED from the lookup tables for that XR at the appropriate phantom age.

### MRI examination

We compared the number of MRI exams received by cases pre- and post-lead implantation to determine if the presence of CIED is related to a reduction in MRI orders. The total number of MRI exams performed in cases vs. controls was also compared. In addition, we performed a review of all indications for CT post-lead implantation in all cases. The patient’s medical records were reviewed to assess the clinical decision-making regarding ordered imaging studies. If an MRI was indicated as the preferred imaging modality based on the clinical objectives, we then evaluated if the CIED resulted in the patient instead receiving a CT exam due to the non–MR-conditional implant. This review was carried out by two cardiothoracic radiologists (A.P. and C.K.R.) with 12 and 22 years of experience, respectively.

### Statistical analysis

We used the Wilcoxon rank sum test (unless otherwise specified) to determine significant statistical difference (*P* < 0.05) in the CED between paediatric CHD patients with CIED and the matched control patients without CIED. To account for the time era of CTs, we performed a comparison (Fisher’s exact test) of the number of CT exams conducted before and after the installation of modern low-dose CT scanners at our institutions (i.e. circa December 2012). Following the expert’s adjudication, we performed a pairwise comparison (Wilcoxon signed-rank test) of CED from actual CT exams received by cases post-lead implant vs. the theoretical CED they would have received if appropriate MRI exams replaced CT in the absence of CIEDs. All statistical analyses were carried out in Python (v3.10.12).

## Results

In this paediatric cohort, we matched 21 among 28 children with epicardial CIEDs (cases) by age at operation, sex, surgical era, and CHD diagnosis to 40 children without CIEDs (controls; *Table 1*; Supplementary data online, *Table S3*). In the remaining seven cases, there were no exact matches, but there were near matches in the control to bracket the major features of the diagnosis. For example, a case with tricuspid atresia 1B with ventricular septal defect (VSD) and no pulmonary hypoplasia was included from 2011 instead of 2009. This decision was made because the diagnosis is rare, and the disparity between the 2009 and 2011 approaches was minimal in our adjudication.

**Table 1:** Patient demographics of CIED patients (*n* = 28) and matched CHD patients with no CIED (*n* = 40)

	Cases, *n* = 28	Controls, *n* = 40
Age at first CIED implant, years		
Mean ± 1 SD	2.1 ± 1.5	
Median (IQR)	2.5 (2.9)	
Follow-up duration, years		
Mean ± 1 SD	8.6 ± 3.9	6.6 ± 4.3
Median (IQR)	8.8 (7.0)	6.2 (6.8)
Male-to-female (%)	46:54	58:42
Summary diagnosis		
Single ventricle physiology	11 (39%)	12 (30%)
AVSD and VSD	7 (18%)	12 (30%)
Tetralogy of Fallot and double outlet right ventricle	5 (18%)	7 (18%)
Transposition of the great arteries	2 (7%)	5 (13%)
Shone complex	2 (7%)	1 (2%)
Ebstein anomaly	1 (4%)	3 (8%)
Indication for pacing		
Surgical heart block	12 (43%)	
Prophylaxis against bradycardia	11 (39%)	
Sinus pauses	2 (7%)	
High-grade AV block	2 (7%)	
Junctional rhythm	1 (4%)	
Device configuration		
IPG and atrial and ventricular leads	16 (57%)	
IPG and ventricular lead only	1 (4%)	
No IPG. Atrial and ventricular leads	2 (7%)	
No IPG. Atrial leads only	9 (32%)	

Numbers in parenthesis are per cent. See Supplementary data online, *Table S3*, for detailed tabulation of case diagnoses.

SD, standard deviation; IQR, inter-quartile range; CIED, cardiac implanted electronic device; IPG, implanted pulse generator; (A)VSD, (atrio)ventricular septal defect; cases, paediatric CHD patients with CIED; controls, paediatric CHD patients without CIED; babygram, whole-body XR.

**Figure 2:**
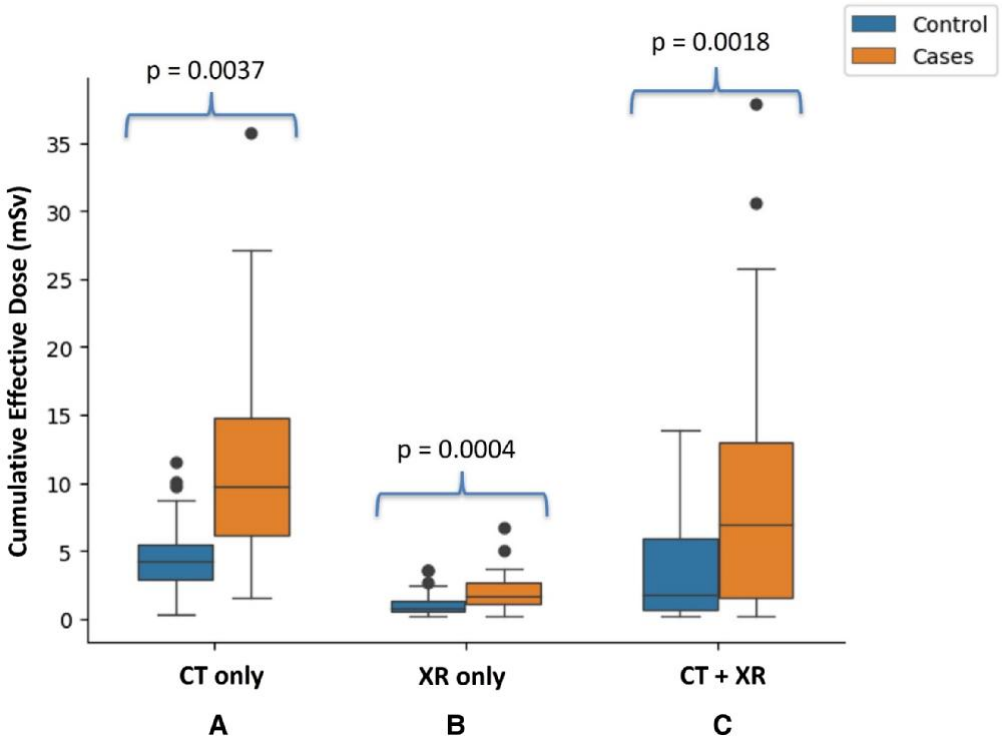
Cumulative ED from CT and XR exams. Box plots of CED for cases and controls from (*A*) CT scans alone, (*B*) XR scans alone, and (*C*) both scans. The median overall CED for cases is 4 × higher than in the controls. In (*A*), the cohort is restricted to cases and controls with at least one CT scan (*n* = 18 and 21, respectively). In (*B* and *C*), all patients in each cohort were used.

The median age at CIED implant in the cases was 2.5 years. There was no significant difference in the median follow-up duration between patients with CIEDs and patients without CIEDs (8.8 vs. 6.2 years, *P* = 0.07, *Table 1*). There was no crossover from case to control (no epicardial lead explants occurred). Cases received four times the ionizing radiation dose from CT and XR than controls (median CED 6.90 vs. 1.72 mSv, *P* = 0.0018, *Figure 2*).

Among patients who received at least one CT exam, the median CED from CT was more than two times higher for cases (*n* = 18) than controls (*n* = 21; 9.72 vs. 4.24 mSv, *P* = 0.0037, *Table 2*). CT exposure accounted for a greater percentage of CED than XR in both cases and controls (79% of CED in cases and 70% in controls). There was no difference in the number of CT exams performed in cases compared with control before the installation of modern low-dose CT scanners at our institutions [24 (41%) vs. 14 (35%), *P* = 0.675].

**Table 2:** CED for CT and XR scans for paediatric CHD patients with and without CIED

CT			XR		
	Cases	Controls		Cases	Controls
*n* (% of total)	18 (64%)	21 (53%)	*n* (% of total)	28 (100%)	40 (100%)
CED (mSv)			CED (mSv)		
Mean ± 1 SD	11.84 ± 9.17	4.77 ± 3.03	Mean ± 1 SD	2.01 ± 1.42	1.05 ± 0.84
Median (IQR)	9.72 (8.62)	4.24 (2.63)	Median (IQR)	1.72 (1.53)	0.73 (0.76)
Age at first CT, years			Age at first XR		
Mean ± 1 SD	1.4 ± 3.2	1.4 ± 3.0	Mean ± 1 SD, years	0.3 ± 0.9	0.3 ± 1.2
Median [IQR]	0.3 (0.5)	0.1 (0.6)	Median (IQR), days	0 (17)	1 (73)
Male-to-female (%)	39:61	52:48	Male-to-female (%)	46:54	58:42
Exams			Exams		
Head	20	7	Single chest (AP)	1659	1100
Sinuses		2	Chest (AP and lateral)	266	146
Spine	4	1	Single full-body (AP)	123	157
Chest	27	22	Full-body (AP and lateral)	9	6
Heart	7	5	Single abdomen (AP)	77	25
Abdomen & Pelvis	1	3	Abdomen (AP and lateral)	20	19
			Others^a^	64	14
Total CT exams	59	40	Total XR exams	2218	1467
(Avg. per person)	(3.3)	(1.9)	(Avg. per person)	(79.2)	(36.7)

^a^Not included in CED

The median CED from XR was more than two times higher for cases (*n* = 28) than controls (*n* = 40; 1.72 vs. 0.73 mSv, *P* = 0.0004, *Table 2*). XR exams of the chest, full body, and abdomen contributed 97% of the total number of XR exams in each group. Therefore, the CED comparison was limited to these three examinations to simplify computation and reduce estimation error.

**Figure 3:**
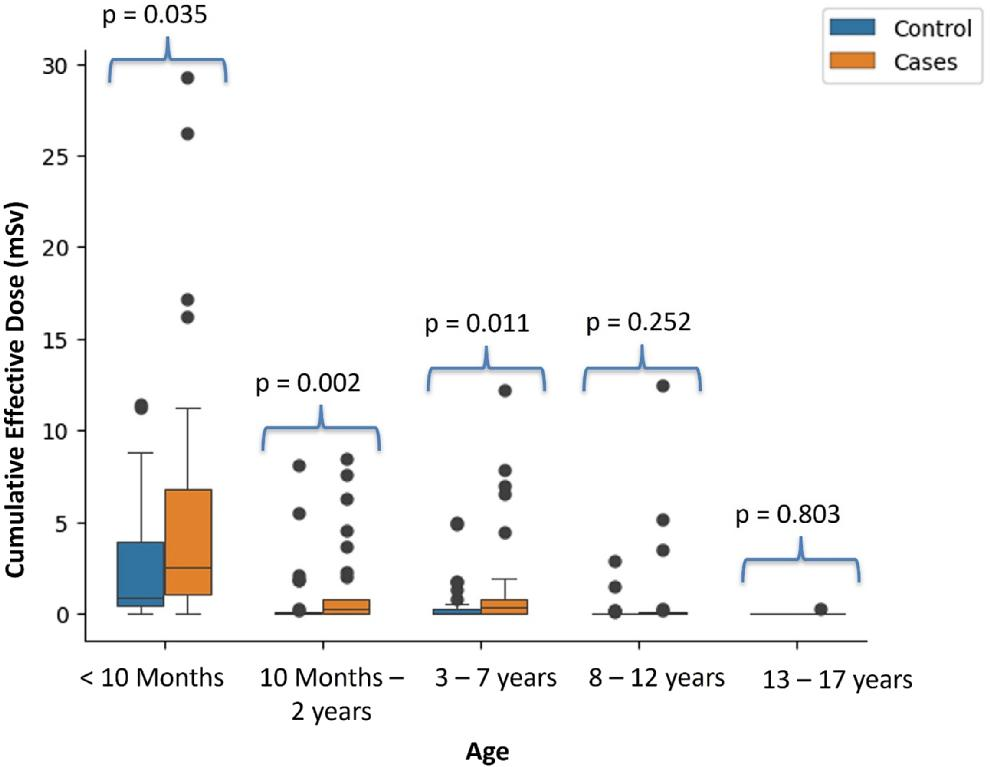
Cumulative ED for paediatric patients by age. Box plots of total CED from CT and XR per age group for paediatric CHD patients with CIED (cases) and paediatric CHD control patients, highlighting when radiation exposure is greatest across lifespan. For this sub-analysis, CED does not carryover across age groups.

Most of the radiation exposure for cases and controls occurred in the first three age groups (birth through age 7), and almost no radiation exposure occurred in the last two age groups (*Figure 3*). Cases received 61% of their total CED before 10 months of age, compared with controls, who received 70% of total CED during the same period. The CED for cases and controls were significantly different (*P* < 0.05) for age groups <10 months, 10 months–2 years, and 3–7 years, whereas there was no significant difference between case and control groups (*P* > 0.25) for the remaining two older age groups (8–12 and 13–17 years).

Cases received 23 MRI exams prior to lead implantation (*n* = 14), but only 1 case had an MRI post-lead implantation (2 exams in the same patient; *Table 3*). Control patients received 31 MRI exams (*n* = 12). The expert adjudication of CT exams post-CIED implantation showed that 24 of the 30 CT exams (80%) for 13 patients would have been eligible for MRI in the absence of CIED (*Table 4*). In the absence of a CIED, MRI would have been a viable alternative for all CT exams of the head, heart, and spine. The median [inter-quartile range (IQR)] CED (*n* = 13) was significantly higher for the actual post-lead CT exams than the theoretical calculated CED if MRI replaced CT in the absence of CIEDs [5.80 (7.51) vs. 0 (1.78) mSv, *P* = 0.0022). In aggregate for this subgroup, the total ED was 93.1 mSv for the actual post-lead CT exams compared with just 18.5 mSv for the theoretical case where 80% of these exams were instead performed with MRI. On average for each patient, CIED presence resulted in a five-fold increase in ED due to the non–MR-conditional implants. In general, these exams represented 35.5% of radiation dose from all CT exams pre- and post-lead implant in patients with epicardial CIEDs.

**Table 3:** Numbers of MRI and CT exams pre- and post-lead implantation for CIED patients compared with non-device patients

CT			MRI			
	Cases			Cases		Control
	Pre-lead implant	Post-lead implant		Pre-lead implant	Post-lead implant	
*n* (% of all patients)	13 (46%)	13 (46%)	*n* (% of all patients)	14 (50%)	1 (4%)	12 (30%)
Exams			Exams			
Head	7	13	Brain	10	1	18
Spine	3	1	CVMR	9		9
Chest	15	12	Spine	4	1	2
Heart	3	4	Appendicitis			1
Abdomen and pelvis	1		Body			1
Total CT exams	29	30	Total MRI exams	23	2	31

CVMR, cardiovascular MRI and includes heart, chest, and magnetic resonance angiography.

**Table 4:** The number of post-lead CT exams and the corresponding effective dose for actual (cases) and theoretical scenario, in which appropriate MRI replaces CT in the absence of CIEDs

	CT scans after CIED, actual		CT scans after CIED, adjudicated (if all CIED patients were MR-eligible)	
Exam type	Number of exams performed	ED (mSv)	Number of CT exams still required	ED (mSv)
Head	13	30.8	0	0
Spine	1	0.5	0	0
Chest	12	39.0	6	18.5
Heart	4	22.7	0	0

## Discussion

Adjudication by two experienced paediatric radiologists showed that 80% of the CT exams post-implantation would have been performed with MRI in the absence of a CIED. This resulted, on average, a five-fold increase in ED from post-lead implant CTs. Paediatric CHD patients with non–MR-conditional epicardial CIED received four times the CED compared with the control group. The higher dose has multiple explanations, including that the presence of a CIED is a marker for more severe disease, thus requiring an increased number of imaging studies. Regardless of causality, our most clinically important finding is that CIED patients received high levels of radiation from medical imaging tests.

Higher radiation exposure for CIED patients has important clinical ramifications. Extensive previous work demonstrates that children with higher radiation have a higher cancer risk. For example, cancer risk was found to be twice as high in CHD patients—ages ranging from birth to 41 years—than in matched healthy controls.^29^ In another study involving over 24 000 adult CHD patients, the cumulative cancer incidence was 15.3%, which correlated with increased ionizing radiation exposure from cardiac procedures.^30^ Such findings highlight the lifelong cancer risk for paediatric CHD patients who are exposed to ionizing radiation early in their care.^31,32^ Our study shows that paediatric CHD patients with CIED may be at greater risk when compared with matched CHD patients with no CIED. As such, radiation dose optimization as well as increased access to non-ionizing medical imaging tests such as MRI could help mitigate radiation burden in this vulnerable patient population.^33,34^ Our recommendation on dose optimization is aligned with the Image Gently statement, ‘…to encourage informed imaging to achieve appropriate study quality at the lowest achievable dose’ in children with CHD.^31^

In our study, CT exams contributed >70% of the CED in our CHD patients. This finding is consistent with earlier publications demonstrating that imaging procedures such as CT and cardiac catheterization were the most important contributors to radiation risk in CHD patients.^35^ For example, in a study by Johnson and colleagues^35^, cardiac catheterization and CT exams represented ∼3% of the total number of examinations but accounted for 81% of the CED in children with heart disease. Those numbers were remarkably similar in our study. For cases, CTs were numerically only 2.7% of all studies but contributed 76% of the total CED (*Table 2*). Our retrospective study included CT data from both older generation and modern low-dose CT scanners. While the total CED in cases would be lower if only the low-dose CT scanners are considered, the relative difference in CED between cases and controls remains.

An additional new contribution of our study is that nearly all diagnostic radiation in this population occurred prior to age 8. These data are complementary to the well-established observation that surgical interventions are clustered in infancy and early childhood in most life-threatening structural CHDs and respond directly to recent calls for comparative metrics in the scientific position statement focused on children from the Image Gently Alliance.^31^ While all retrospective studies are subject to selection bias, our data suggest that in this population, clinical efforts to decrease radiation exposure in CHD patients with CIEDs should focus on infants and young children.

We found through expert adjudication that up to 80% of the CTs obtained in cases after CIED implantation could have been imaged using MRI if either no CIED had been present or if CIED-tolerant MR protocols had been in place. The implication of this is that CT was chosen because of the presence of CIEDs. Our data suggests that CT scans in CIED patients represent an opportunity to mitigate risk by considering the risk–benefit rewards of MRI in children with CIEDs, whether devices are MR-conditional or non–MR-conditional. This risk–benefit approach is consistent with the recent 2021 PACES Expert Consensus Statement.^12^ While it might be possible to replace cardiac MRI with an echocardiogram, evaluating intra-cardiac anatomy or morphology necessitates combining an echocardiogram with catheterization. In addition, ultrasound evaluations of the brain and spine are largely dependent on the pathology and primarily suitable for infants up to 2 to 8 months of age.

To increase the utilization of MRI in children with epicardial CIED, future research priorities must include decreasing MRI safety risk by developing lower-risk configurations for non–MR-conditional CIED systems^36,37^ and improving MRI diagnostic yield by implementing strategies to reduce image artefacts caused by CIEDs.^38–40^ Moreover, MRI is also under-utilized in the paediatric population due to long scan times and complexities in scanning.^41^ For instance, cardiovascular MRI images are acquired under breath hold to minimize artefacts due to respiratory motion. Self-controlled breath holds are often not achievable in children, and general anaesthesia is often necessary for children younger than 7–10 years old.^34,42^ Anaesthesia is a particular concern for children < 5 years old because it may result in neurodevelopmental issues.^43^ Other technical barriers to optimal image quality in young CHD patients include small hearts, fast heart rates, and complex anatomy. Therefore, it is also important to consider highly accelerated,^38,39,44^ free-breathing techniques to alleviate the need for general anaesthesia as well as volumetric techniques with high spatial and temporal resolution, to increase the benefit of MRI in this patient population .

### Limitations

This study has several limitations. First, too few data are available for us to fully model CIED impact on the dose distribution within the ICRP reference phantoms. When impinged on by radiation, metal surfaces (such as the CIED) are known to release secondary electrons that contribute to dose build-up or backscatter radiation.^45,46^ Therefore, the CED calculation performed with the ICRP phantoms in the NCI dosimetry software is conservative for paediatric CHD patients with CIEDs. The actual CED our cases received may have been higher than modelled due to dose build-up and backscatter.

Secondly, the current implementation of the NCICT software does not provide scanner-specific automatic tube current modulation algorithm and is limited for retrospective CT data without slice-specific CTDI_vol_ or mAs.^22^ Therefore, tube current modulation was not included in the CED calculations, which may likely decrease the dose in every patient in this study.^47^

Thirdly, historical patient-level exposure information in fluoroscopic modalities was limited and did not provide sufficient information to model CED. Radiation dose specifiers such as DAP, cumulative air kerma, or peak skin dose were not reported in the majority of studies. Although fluoroscopy time was reported in more than 50% of patients, it is not sufficient to estimate radiation dose from these exams.^48^ In addition, we did not include nuclear imaging tests in this retrospective study, because only a few (four cases and one control) patients received them.

Fourthly, we prioritized matching criteria including age at operation, sex, surgical era, and CHD diagnosis to minimize the discrepancy in care due to variations in medical and surgical approach. However, the careful matching decreased our total numbers (simple surgeries rarely result in patients with CIEDs) but still does not necessarily guarantee that the two groups were equivalently ‘sick’. The higher number of XRs for the CIED group may be an indication that these patients had longer hospital stays and/or higher acuity illnesses than matched controls. It is therefore possible that the detectable differences in ED between the cases and control groups may be driven by differences in the total number of exams between the two groups . Nevertheless, the main takeaway from our findings remains the same—that CIED patients are exposed to high levels of CED and there is an urgent need for strategies to mitigate radiation exposure from medical imaging tests in children with CIEDs.

## Conclusion

Nearly 80% of the CT exams likely could have been performed with MRI in the absence of a CIED. Paediatric CHD patients with CIED received four times higher CED than matched patients with no CIED. Increased access to medical imaging tests without ionizing radiation such as MRI could help mitigate the radiation burden in this vulnerable patient population.

## Supplementary Material

qyae060_Supplementary_Data

## Data Availability

All data supporting the findings of this study are available within the paper and its Supplementary data.

## Lead author biography

Oluyemi Bright Aboyewa is a PhD candidate at Northwestern University in the Biomedical Engineering Department. His rese arch focuses on cardiovascular MRI in paediatric patients under Dr. Daniel Kim in the Radiology Department. Oluyemi holds a master’s degree in medical physics with backgrounds in medical imaging, radiation therapy, radiation dosimetry, and protection. He is passionate about improving patients’ treatment outcome with MRI guidance.
