# Supplementary material for: Cumulative radiation dose from medical imaging in paediatric congenital heart disease patients with epicardial cardiac implantable electronic devices
Source: Eur Heart J Imaging Methods Pract. 2024 Jun 13;2(1):qyae060. doi: 10.1093/ehjimp/qyae060 (PMC11251694; doi:10.1093/ehjimp/qyae060)
Supplement: qyae060_Supplementary_Data [file qyae060_Supplementary_Data.pdf]

Supplemental Material

Supplemental Methods

There are five defined age groups (See Methods). For the purpose of the Supplemental Tables, they are labeled in this way:

0: < 10 months

1: 10 months - 2 years

5: 3 – 7 years

10: 8 – 12 years

15: 13 – 17 years

The dose product average (DAP) for the five phantom age groups was calculated as average of DAP from the DICOM of 10 patients randomly selected from our database who had a single view anteroposterior (AP), and two views AP and lateral XRs of full torso, chest, and abdominal exams. We selected the half value layer (HVL) in the NCIRF software that closely matches the average HVL (mm Al) at different tube potentials for the x-ray units at our institution.

Table S1: XR NCIRF parameter table with the calculated effective dose for the five-age group male and female pediatric phantoms.

Anteroposterior			Isocenter							Effective Dose (μSv)	
**X-ray Procedure**	**Body Landmark**	**Age (Years)**	**X** (cm)	**Y** (cm)	**Z** (cm)	**FOV (H X W)** (cm^2)	**KVP** (V)	**HVL** ($mm\ AL$)	**DAP** ($dGy \cdot cm^2$)	**Male**	**Female**
Single View Chest	Mandible to End of Rib cage	0	12.60	8.00	31.70	13×14	60	2.25	0.104 ± 0.037	24.60	25.50
		1	19.80	8.80	52.70	21.5×20	60	2.25	0.170 ± 0.066	15.40	14.30
		5	26.50	10.60	82.50	27×28.5	70	2.61	0.204 ± 0.092	11.30	11.50
		10	34.90	12.00	107.50	36×34.5	70	2.61	0.469 ± 0.207	14.00	13.80
		15	44.60	16.00	131.40	40×42.5	80	3.01	1.359 ± 0.408	18.40	22.30
Two Views Chest	Mandible to End of Rib cage	0	12.60	8.00	31.70	13×14	70	2.61	0.184 ± 0.062	49.20	50.00
		1	19.80	8.80	52.70	21.5×20	90	3.38	0.200 ± 0.052	22.70	22.50
		5	26.50	10.60	82.50	27×28.5	90	3.38	0.435 ± 0.107	27.40	27.70
		10	34.90	12.00	107.50	36×34.5	90	3.38	0.572 ± 0.294	19.10	19.50
		15	44.60	16.00	131.40	40×42.5	110	4.11	0.911 ± 0.288	14.70	17.60
Full-torso plain film imaging, 1 view	Mandible to Pelvis	0	12.60	8.00	26.00	24×14	60	2.25	0.155 ± 0.032	27.40	29.80
Full-torso plain film imaging, 2 views	Mandible to Pelvis	0	12.60	8.00	26.00	24×14	60	2.25	0.235 ± 0.089	39.7	38.6
Single View Abdomen	Mid heart to Pelvis	0	12.60	8.00	22.50	17×14	60	2.25	0.151 ± 0.041	27.00	28.70
		1	19.80	8.80	41.10	23×20.5	60	2.25	0.298 ± 0.086	27.10	30.00
		5	26.70	10.60	65.20	32×24	70	2.61	1.390 ± 0.731	66.10	76.50
		10	34.80	12.00	84.20	42×29.5	70	2.61	2.636 ± 1.010	58.50	66.90
		15	44.60	16.00	102.70	50×37.5	80	3.01	11.624 ± 9.849	153.00	181.00
Two Views Abdomen	Mid heart to Pelvis	0	12.60	8.00	22.50	17×14	60	2.25	0.135 ± 0.065	23.50	26.90
		1	19.80	8.80	41.10	23×20.5	60	2.25	0.299 ± 0.067	29.40	32.60
		5	26.70	10.60	65.20	32×24	70	2.61	1.247 ± 1.039	60.00	68.60
		10	34.80	12.00	84.20	42×29.5	70	2.61	3.388 ± 1.372	74.50	86.90
		15	44.60	16.00	102.70	50×37.5	80	3.01	7.069 ± 4.795	92.10	111.00

Table S2: XR NCIRF parameter table with the calculated effective dose for the five-age group male and female. pediatric phantoms

**Lateral**			**Isocenter**							**Effective Dose (μSv)**	
**X-ray Procedure**	**Body Landmark**	**Age (Years)**	**X** (cm)	**Y** (cm)	**Z** (cm)	**FOV (H X W)** (cm^2)	**KVP** (V)	**HVL** ($mm\ AL$)	**DAP** ($\mathrm{dGy} \cdot \mathrm{cm}^2$)	**Male**	**Female**
Two Views Chest	Mandible to End of Rib cage	0	12.60	8.00	31.70	13×11	70	2.61	0.177 ± 0.087	31.40	27.60
		1	19.80	8.80	52.70	21.5×18	90	3.38	0.210 ± 0.065	19.90	20.00
		5	26.50	10.60	82.50	27×18.5	90	3.38	0.411 ± 0.095	19.30	19.90
		10	34.90	12.50	107.50	36×22.5	90	3.38	0.962 ± 0.350	23.50	24.40
		15	44.60	16.00	131.40	40×26.5	110	4.11	2.637 ± 0.811	33.70	45.00
Full-torso plain film imaging	Mandible to Pelvis	0	12.60	8.50	26.00	24×12.5	70	2.61	0.239 ± 0.099	30.90	32.00
Abdomen	Mid heart to Pelvis	0	12.60	8.50	22.50	17×12.5	60	2.25	0.160 ± 0.041	22.60	24.80
		1	19.80	8.70	41.10	23×17.5	70	2.61	0.397 ± 0.217	27.50	33.80
		5	26.70	11.00	65.20	32×18.5	70	2.61	1.675 ± 1.240	56.70	61.90
		10	34.80	12.70	84.20	42×21.5	80	3.01	4.410 ± 1.682	70.80	75.80
		15	44.60	16.00	102.70	42×27.5	80	3.01	10.283 ± 7.865	110.00	109.00

Table S3: Detailed anatomic diagnosis for all cases (N=28).

Diagnosis	Number of patients with CIED
Hypoplastic right heart syndrome (pulmonary atresia and intact ventricular septum)	3
Hypoplastic right heart syndrome (tricuspid atresia and ventricular septal defect)	3
Atrioventricular septal defect	2
Perimembranous ventricular septal defect	2
Tetralogy of Fallot with pulmonary atresia	2
Transposition of the great vessels with ventricular septal defect	2
Coarctation of the aorta with ventricular septal defect	1
Double outlet right ventricle	1
Ebstein anomaly	1
Ebstein anomaly with hypoplastic right heart syndrome	1
Hypoplastic left heart syndrome (mitral and aortic atresia)	1
Hypoplastic left heart syndrome (mitral stenosis and aortic atresia)	1
Hypoplastic right heart syndrome (tricuspid atresia and restrictive ventricular septal defect)	1
Interrupted aortic arch with ventricular septal defect	1
{S,L,L} transposition of the great vessels with left-sided atrioventricular valve atresia	1
{S,L,L} transposition of the great vessels with double outlet right ventricle	1
{S,L,I} transposition of the great vessels with double outlet right ventricle and pulmonary stenosis	1
Shone complex	1
Subvalvar mitral stenosis	1
Transitional atrioventricular septal defect with primum ASD and restrictive VSD	1
**TOTAL**	**28**

All patients had [S,D,S] segmental anatomy unless otherwise noted.

Supplemental Figure

(A)

(B)

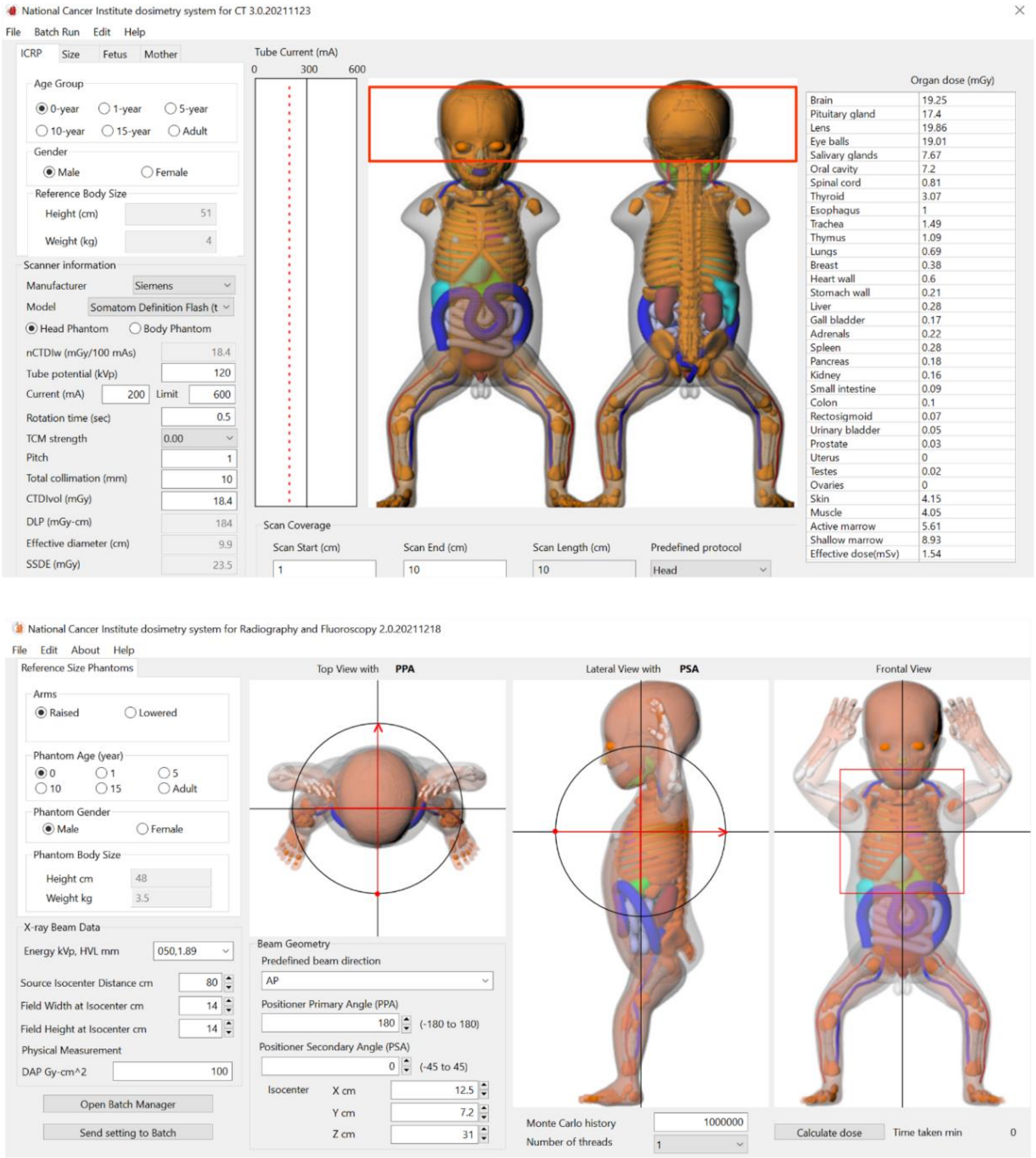

Figure S1: (A) NCICT software user interface for CT effective dose calculation with ICRP pediatric phantoms. (B) NCIRF software user interface for XR effective dose calculation with ICRP pediatric phantoms.
